# Students' Performances in Computer Programming of Higher Education for Sustainable Development: The Effects of a Peer-Evaluation System

**DOI:** 10.3389/fpsyg.2022.911417

**Published:** 2022-09-13

**Authors:** Tsung-Chih Hsiao, Ya-Hsueh Chuang, Tzer-Long Chen, Chien-Yun Chang, Chih-Cheng Chen

**Affiliations:** ^1^School of Arts, Southeast University, Nanjing, China; ^2^Department of Finance, Providence University, Taichung, Taiwan; ^3^Department of Healthcare Administration and Medical Informatics, Kaohsiung Medical University, Kaohsiung, Taiwan; ^4^Department of Fashion Business and Merchandising, Ling Tung University, Taichung, Taiwan; ^5^Department of Automatic Control Engineering, Feng Chia University, Taichung, Taiwan

**Keywords:** technical education, sustainable education, peer review, graphical user interface, block-based computer programming language logic design

## Abstract

Modern education attaches great importance to interdisciplinary skills, among which computational thinking is a core element, and heralds a new era. IT application has shaped education in the 21st century. Computational thinking has provided further impetus for building an all-encompassing social network and fostering a DIY culture enabled by digital technologies. One empirical study used four apps to test children's development in computational thinking and fluency. The article will help students overcome their fears of coding. Peer reviews provide students with an opportunity to learn from each other and become more motivated. These reviews also serve as feedback for teachers to evaluate students' performance. Experimental design is used in this study, and a peer review system is implemented. Freshmen attending a programming class in a university are used as samples. At the class, students write computer programs with f-Chart, which provides a graphical user interface for students to learn programming logic and design. Zuvio, a cloud-based interactive response system, is used to conduct the peer reviews. The data of this study are analyzed through R. The results show not only an improvement in students' learning performance but also a gap between students' peer review scores and teachers' evaluation scores. Learning feedback and evaluation is crucial to transform education between students and teachers into a sustainable cycle system.

## Introduction

Learning styles and performance assessment have been evolving with time. Traditional teaching strategies put higher educational institutions in a tight spot to engage students' interests and improve performance. Information technology (IT) has become a key driver of global economic growth, especially for sustainable education. Students must cultivate computational thinking by learning computer programming language in order to meet the need for IT development. The capability of computer programming language is one of the basics for IT development. However, students often have fears and difficulties in learning computer programming language. Performance in learning computer programming language logic is studied in this research. The scores of midterm and final examinations are used to evaluate the improvement of students' learning performances. The sustainability of the development of computer programming language will be a future issue.

The four important aspects of learning are learner-centered, knowledge-centered, assessment-centered, and community-centered. “Assessment-centered” could be viewed as the central part in the learning process. If learners and teachers underestimate the assessment, it will have a considerable gap between the instruction and learning.

By contrast, traditional assessment techniques and teacher–student relationship put enormous pressure on students to such an extent that they lose enthusiasm for learning. On the other hand, if instructors use peer review as feedback, it will make teacher–student relationship more productive. The problem in the past was the influence by Asian cultural background because many problems and obstacles in learning were caused from learner's own social pressure. Instruction nowadays should change to student-centered.

The research object of this study is college freshman learners of information science. Whether they are beginners or have background knowledge of the course in high school is taken into account. They were divided into experimental groups to teach by peer review. The self-made results were reported during the semester examination. Peers and teachers are allowed to grade; the control group uses traditional assessment to teach, and test papers are distributed during the semester examinations, so that students can answer and test based on the test questions, and then the teachers review the grades.

The literature review of this study focuses on difficulties in learning programming logic, equation programming, introduction to interactive instruction platforms, and further reading.

## Literature Review

To address this challenge, universities have introduced campus-wide programming courses. However, intimated by the English-language user interface and programming language, many students tend to lose confidence, lack motivation to complete the course, etc. (Calder, 2010; Chen et al., 2020). Thus, this study employed the block programming interface to improve the learning environment of programming, stimulate students' interest in programming, and improve students' impression that programming is difficult. The teaching model employed in the study encouraged student interaction and discussion, cultivating the learning atmosphere that allows peer discussion and work sharing. This teaching model can also enhance students' abilities in expression, problem-solving, and promoting group cooperation. Through flow chart programming, block programming interface allows students to focus the programming learning on understanding programming logic. At the completion of programming, students can present their works on mobile phones. In addition, this programming approach can also control hardware devices, increasing the richness of the outcome and enhancing students' interest in learning programming. When evaluating students' learning outcomes, this study employed peer evaluation for mutual evaluation, encouraging students to analyze the strengths and weakness of other students' works to have further understanding of their own works. Peer scoring and teacher scoring were used to analyze the research results to examine whether student achievement, learning proficiency, and learning enthusiasm have improved.

Information education is an integral part of modern higher education. It is worth mentioning that this form of education is not to spoon-feed students with programming syntax but to teach them how to think like a programmer and develop creative thinking to solve problems (Calder, 2010; Finn and Garner, 2011; Hillsdale et al., 2019; Ion et al., 2019; Hickey et al., 2020). The focus of promoting programming education is to develop and guide students' programming logic ability. In the past, students' learning aims in programming courses were command interface. For students who are new to programming, it is relatively difficult, which caused students to have no interests in programming at all (Kordaki, 2012). In order to arouse students' interest, we must understand students' bottlenecks in learning programming (Liu et al., 2018a). After investigation, there are three steps that can arouse students' interests in programming. The first step is that students need to immediately display their self-designed programming works, such as apply communication to control automatic mobile vehicles and robots, experience how to use block program designing, and control designing to solve problems in order to understand the application and fun of programming (Liu et al., 2018b). The second step is to simplify the interface of program coding, and the third step is to train students' problem-solving ability (Luaces et al., 2018) to keep students motivated as a sustainable development goal. By applying block program design, students are taught to learn program designing to solve problems and design related information function applications and integrate other areas to create innovative applications. While students work with their peers in the practical application of the topic, their independent thinking abilities are also developed (Lundstrom and Baker, 2009). Instruction means granting students to obtain specific abilities and skills. Therefore, how to make good use of evaluation methods to help learners is the ultimate sustainable development goal of instruction, and it is also a significant issue for educators in higher education to care about (Mine and Colin, 2018).

In recent years, peer review and self-evaluation have been receiving growing attention on how to better motivate students, improve their performance, foster a supportive learning environment, and develop sustainable education (Murillo-Zamorano and Montanero, 2018).

Peer review can be conducted through group discussions to help students forge closer bonds, develop critical thinking, and improve learning efficiency in the process. Past research has found that peer evaluation provides instant feedback among students, which can foster them the ability of solving problems and improve their leaning performance (Nielsen, 2014). In the process of problem-based learning, students will actively seek out problems, realize the core of problems, and then solve problems, that is, using peer evaluation could get rid of the dilemma of the problem-solving. In the peer learning mode, students will advance a conscious attitude to their own learning to promote and deepen what they have learned.

### Block Programming

The purpose of information education is to enable students to learn how to solve problems through applying information. Therefore, the purpose of teaching students programming is not to make them memorize programming language but to make them learn how to solve problems by applying programming logic (Calder, 2010; Liu et al., 2018a). According to extant investigations, there are three keys to inspire students in learning programming (Kordaki, 2012; Hillsdale et al., 2019). First, in the process of programming, students can view instantly the output of the programming, for example, using a program to control a robot or an automatic mobile vehicle; students can see immediately the result of the robot being controlled by the program they have just accomplished or modified. Second, the software interface for programming is based on graphic, which makes it easier for students to understand. Third, guiding students' thinking logic in writing programs and allow students to learn how to write a complete program step by step. Compared to the traditional programming language development interface, block programming can better meet the aforementioned three keys. Students can focus on thinking the logic of programming, and it can stimulate students' interests in learning programming. Thus, it is a language more applicable for beginners learning programming (Piteira and Costa, 2013; Luaces et al., 2018; Papadakis, 2018; Papadakis and Orfanakis, 2018; Ion et al., 2019; Papadakis and Kalogiannakis, 2019; Ladias et al., 2021, 2022).

### Self-Assessment

In the process of learning, self-assessment allows students to learn the extent of their own growth. Research has found that student self-assessment is more effective than teacher evaluation to improve student learning outcome (Kordaki, 2012). Nielsen (2014) believed that self-assessment not only helps students build self-confidence and find their own weaknesses but also enhances learning motivations, which bring positive impacts on learning performance (Liu et al., 2018b). Self-assessment can guide students develop their own learning strategies to improve learning effectiveness (Lundstrom and Baker, 2009).

The recommendations from the extant studies on applying self-assessment in teaching are as follows (Liu et al., 2018b):

(1) Prior to peer assessment, the teacher should inform students the assessment approach and criteria in detail.
(2) Students should have a clear understanding of the purpose of self-assessment. The teacher should advise students on how to complete the self-assessment and then give a score based on students' written reports.
(3) Students should participate in establishing assessment criteria, which would help them understand the criteria and thus conduct peer assessment accordingly.
(4) When assessing students' work, teachers should emphasize on the students' development process and focus on giving positive suggestions, which would trigger students' learning motivation.
(5) Self-assessment is a formative assessment, rather than a summative assessment. Teachers should not over-emphasize the importance of self-assessment scores, instead should focus on the process of assessment, allowing students to self-assess in an honest manner.
(6) Teachers should provide feedback on the self-assessment of students.
(7) The students' self-assessment should include the assessment on both the overall performance and detailed items.
(8) Students' self-assessment can be incorporated with peer feedback, allowing students to know how to correct and improve their works.

Self-assessment allows students to point out the strengths and weaknesses of each other's work, inspire their learning motivations, and understand their own drawbacks (Mine and Colin, 2018).

### Peer Evaluation

Through peer evaluation, students could increase their chances of interaction with their peers, understand how to modify their works with the peers' suggestions, and defend their own ideas and answers to the questions, which will significantly enhance students' problem-solving capacity (Liu et al., 2018b; Mine and Colin, 2018; Murillo-Zamorano and Montanero, 2018). The social identity theory is an established theoretical framework founded in psychology that promotes understanding in participation within sciences and engineering, including computer programming (Roy, 2012). However, teachers must plan carefully when setting scoring criteria to avoid reducing students' interest in learning (Nielsen, 2014). When using peer evaluation, students' learning motivation and pressure are from other students, which in turn can reduce the opposition between teachers and students (Seifert and Feliks, 2019). However, when conducting peer evaluation, students should focus on their feedback to other students on the improvement of the works, and not general comments (Nielsen, 2014). Thus, to allow peer evaluation to function properly, Finn and Garner (To and Panadero, 2019) made the following suggestions to teachers:

(1) Explain the content and procedure of feedback for peer assessment: The teacher should plan the scope of assessment and explain how it will be preceded to students. When the content of feedback is not clear, students may not under-stand the feedback content and thus unable to provide adequate feedback to improve the student works. Thus, teachers should assist and guide students to clearly express their feedback content so that students can promote their learning outcome through peer assessment.
(2) Provide students with support and encouragement: The teacher should provide support in the learning and emotional aspects of the feedback content (Liu et al., 2019). In the learning aspect, the teacher should assist students in explaining the content of feedback, allowing the assessed students understand how to modify the work (Liu et al., 2022). In the emotional aspect, students should be encouraged to give positive response toward the peer feedback.
(3) Pay attention to the negative feedback students gave: The peers' negative feedback may lead to the resistance of the assessed students, and they may refuse to modify the work following the feedback (Liu et al., 2016). Thus, the teacher should encourage students to, from the perspective of helping their peers grow, take feedback with a positive attitude.
(4) Examine students' learning problems: Teachers should confirm students' learning status through the feedback from peer assessment and adjust their teaching content accordingly.
(5) Discuss the inconsistent views with students: Students may hold opposite perspectives in their feedback. Teachers should assist students through further discussions and integrate their opinions into a summarized feedback with different perspectives.
(6) Based on the aforementioned suggestions, the role a teacher plays in peer evaluation is to guide students in the student assessment system, providing timely assistance when students encounter difficulties so that peer assessment can improve students' learning outcome.

### Interactive IT in Classroom

Effective usage of IT can promote the learning outcome of classroom learning. The other theoretical framework used to guide this study is self-efficacy. Self-efficacy is defined as persons' personal beliefs that they can exhibit behaviors necessary to act or perform in a specific role (Roy, 2012). The IT used in this study is Zuvio. Zuvio allows teachers to ask questions *via* mobile phones and immediately get students' answers in the class. There are two ways to use Zuvio: first, students can download and install Zuvio app in their mobile phones and answer the teacher's questions, and second, without installing Zuvio app, students can scan the QR code through their mobile phone, go to the webpage, and answer the questions. Students' answers will be automatically summed up, and a chart will be generated to allow teacher to view the correct rate of the students' answers to the questions. In addition, Zuvio also provides the function of peer-to-peer assessment, which allow the export of students' answers. The interactive IT in classrooms can increase students' classroom participation and promote peer interaction to enhance student learning outcome.

Zuvio enables the instructor and students to engage in real-time interaction, which gives simultaneous feedback. Apart from the online application, this software can be integrated with offline courses to meet both instructors' and students' needs, that is, using innovative techniques to facilitate real-time interaction and increase learning effectiveness. To be more specific, Zuvio has four procedures: the cloud test prepared by the instructor before class; the virtual test taken by students; visualized interaction; and quantitative analysis. Real-time checks of students' progress and performance evaluation are examined through anonymous or non-anonymous questions which are tracked by Zuvio for analysis.

### R Language

R can enhance its statistic or drawing functions through suites, for example, Big Data Suite, a suite that networks with other programming languages (Zahedi et al., 2021). In developing R-related apps, R Studio is the most popular integrated development environment (IDE). R Studio can turn data into charts and turn figures into visual graphics, allowing data analyzers to more efficiently understand the meaning of the figures.

The aforementioned literature reveals that the traditional teaching method poses a big challenge to students who have no background knowledge of programming language. Gradually, they lose self-confidence and motivation to complete the course. Learner-friendly solutions have been provided. However, they aim to incentivize students, rather than motivating them to move forward. Although such a method does not spoon-feed students, their learning performance is undermined. Considering that language syntax is the only practice-oriented programming course, it is all the more important to remove learning hurdles and teach students how to think critically and creatively. In fact, this is the fundamental aim of programming education.

Learner-friendly elements to foster computational thinking include natural language description of blocks, drag-and-drop interactions, and modular languages. Compared to traditional, text-based programming, this course is more intellectually demanding, yet it enables learners to have a good grasp of programming logic. Prior research has shown that a new visually enhanced programming environment is more welcoming, thus reducing cognitive load. That explains why a visually enhanced programming environment with a drag-and-drop interface is the most popular tool among beginners. Block equation programming can increase learning effectiveness. An interactive instruction platform is another major plus. Classcraft is clear evidence that technology has great potential for engaging students. Existing research has shown that students' input and attitudes have a positive influence on learning outcomes.

This research proves that the innovative teaching method that combines peer assessment, block-based programming, and interactive instruction platform does increase programming learning outcomes. Thus, the teaching method designed by this research has 2-fold benefits: improving learning performance and developing college-wide programming education.

## Conceptual Framework

**Figure 1:**
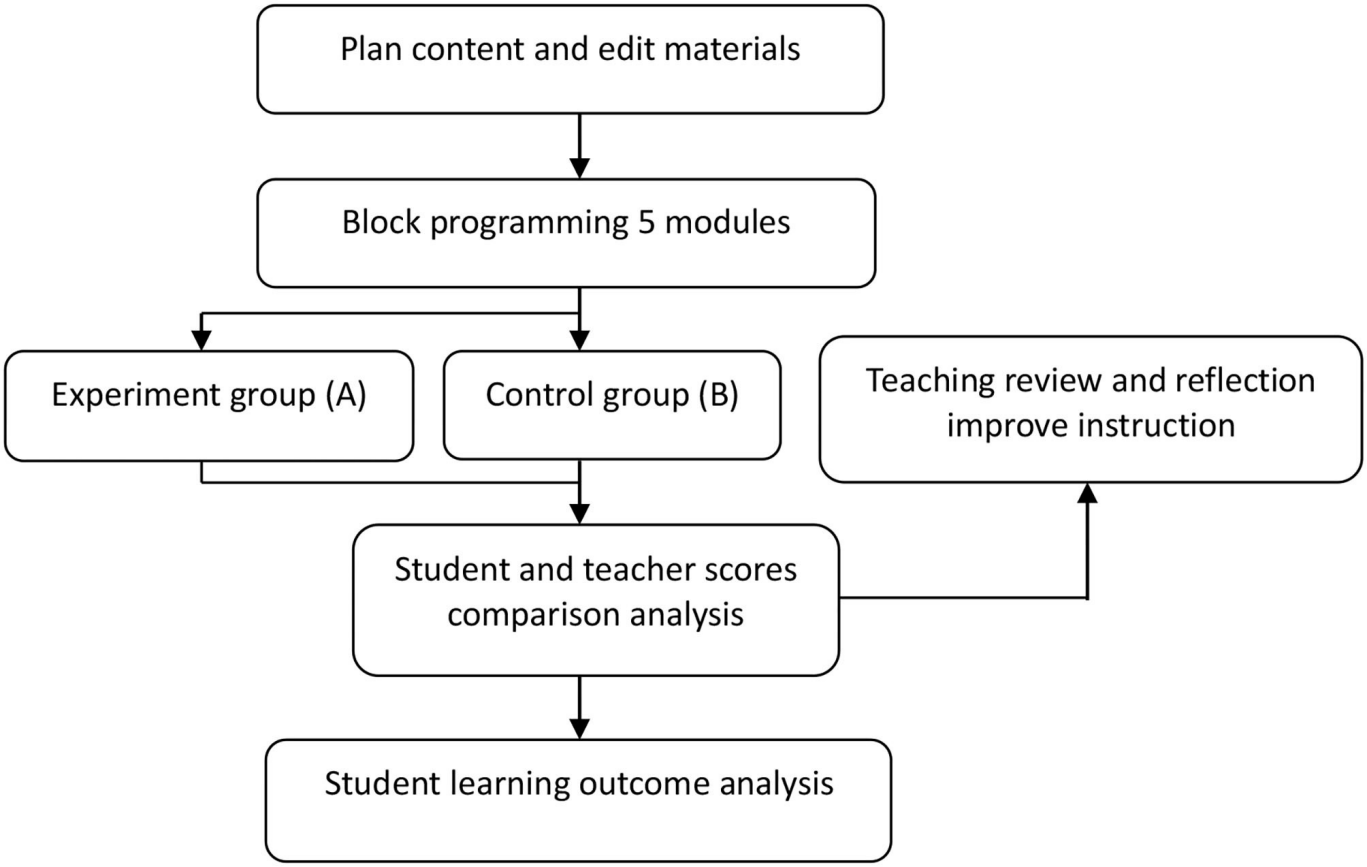
Conceptual framework.

The conceptual framework of the study is shown in Figure 1. It begins with how to plan and write the content and teaching materials for block programming, followed by the instruction of the first five modules, while dividing the class into the peer assessment group (experiment group) and traditional instruction group (control group) to explore whether different instruction models affect students' performance. Next, the students' achievement and assignment-related data will be compared; the teaching review and reflection will be conducted for the second half of the semester to adjust the instruction model accordingly. Finally, the analysis on students' learning outcome will be performed by using the adjusted instruction model.

Figure 1 shows the structure and procedures of this study. It took 10 weeks to develop a module-based course schedule, whose first five units are covered in this research. The following 9 weeks were devoted to the instruction and learning of these five units. The traditional teaching method was applied to this process. An experiment had been run for 2 weeks to compare results reported by the peer assessment group and the controlled one. Both groups were given a test in the same content, but in different orders of questions; 1 week was spent examining the scoring difference between peer assessment and instructor evaluation, followed by reflection and feedback. This led to adjustments to the course schedule and teaching methods for the next program. At the same time, discussions were conducted on how different teaching methods influence homework and achievements. Another 3 weeks were devoted to comparing grades and homework scores. Statistical analysis was conducted to explore the possibility of adjusting the teaching method for the five units before the conclusion of student achievements was drawn.

### Brigade Laboratory Method

It is imperative to understand how to adjust the teaching progress and the content during the course with the students' feedback. In order to understand the learning status of students, assessment is the most direct and specific way in education. It is often used to know whether students have absorbed the content of the classroom. The study targeted the information science-related majors in a basic programming course; the subjects are divided into the experiment group and control group, as shown in Table 1. The experiment group adopted the new teaching model of peer assessment and self-evaluation; the control group adopted the traditional lecture model. After the midterm examination, the first teaching review was conducted, and the curriculum was adjusted accordingly to be followed by the second half-semester of teaching. It was expected that a new teaching model will bring a better learning outcome of programming than the traditional one. The students under new teaching model should outperform the students under traditional teaching model in applying programming in problem-solving and logical thinking.

**Table 1:** Experiment group and control group for research hypotheses.

**School groups**	**Regular/vocational senior high non-information science major**	**Regular/vocational senior high information science major**
Experiment (A Class)	(Peer assessment) (A1) Block programming teaching model	(Peer assessment) (A2) Block programming teaching model
Control (B Class)	(Traditional assessment) (B1) Programming teaching model	(Traditional assessment) (B2) Programming teaching model

### Implementation Steps and Peer Evaluation Rubrics

The subjects of the study were the freshman of information science, and the course was programming. The study employed peer evaluation to calculate the students' grade, allowing students to discover the strength and weakness of self and each other's works to elevate their programming capacity. There were six stages in the evaluation:

(1) Turning in assignment: Students turned in their assignment to the digital learning platform system.
(2) Assignment evaluation: Students scored the assignment by following assessment criteria.
(3) Teacher evaluation: Teacher scored the assignment.
(4) Peer assessment: Students scored the students making the presentation.
(5) Self-assessment: Students also scored their own works while scoring others.
(6) Assignment performance: Upon the completion of peer and teacher assessment, students made corrections based on the feedback.

The traditional evaluation method is divided into two stages:

(1) Examination stage: The teacher issues midterm and final examinations in the classroom.
(2) Teacher rating: Teachers rate students' midterm and final examinations.

This study built the peer evaluation rubrics (Table 2) based on the following principles:

(1) Design the scoring criteria for the course of programming logic.
(2) Build the assessment aspects and define the assessment standard.
(3) The description of the assessment should be easy to understand.
(4) The items and subheadings of assessment standard are clear and precise.
(5) Determine the standards of five levels, for example, excellent, very good, and fair.
(6) Determine the score range of each level.
(7) Calculate the number of operations using the criteria example of the assessment.
(8) Listen to the student feedback and correct accordingly.
(9) Communicate and discuss the new assessment criteria with students and ensure they understand the assessment standard.

**Table 2:** Peer assessment rubric.

**Items**	**Excellent**	**Very good**	**Good**	**Fair**	**Insufficient**
Processing logic	Develop a processing method independently.	Follow thinking pattern of others but implement independently.	Follow processing method of others.	Need others' assistance to implement.	Can not implement to the question.
Component usage	Additional components are added and functional.	The components function to the expectation.	Use excessive components that do not affect functions.	Components used are not functional.	Piece together components inadequately.
Program simplification	The code is concise with additional functions.	The code is streamlined and achieves implementation goals.	The code is not streamlined but achieves implementation goals.	The code functions adequately but can perform setting.	The code file is lengthy and doesn't meet the implementation goals.
Appearance	Use extra appearance components for typesetting.	Design and typeset against the appearance.	The appearance design is moderate with fewer polishing.	Only a small part of appearance is arrayed.	The appearance design is not beautified.
Functionality	Fully functional and include expansional functions.	The function performs correctly and produces results.	The function is normal with occasional unexpected results.	The function is generally normal with frequent unexpected results.	The function can't function and be executed normally.

The virtual test is taken *via* a smartphone or computer whose system is preset with a student roster and test-taking tutorial. When logging on to the cloud testing system, students will receive a notification about the course and its related test. This real-time system allows examinees to remain anonymous, interact with peers, and reflect on lessons learned from the test. As a result, the learners find the course more appealing. It is worth mentioning that before the test, students must familiarize themselves with peer assessment rubrics and Zuvio manual (Table 2).

### Data Processing and Analysis Approach

The study analyzed the distribution of data from the experiment group and control group against teacher's evaluation while making score analysis on the major and non-major groups. To further understand the students' learning progress and the scoring differences between the student and teacher, the study calculated the following two indicators and analyzed the changes in the indicators.

This study examines the performance of 72 students. Before grouping, they answered 10 questions to test foundational knowledge about programming logic. Among the examinees, 38 had some background knowledge, while the rest 34 had none. The former scored 6.78 points, and the latter 3.56, a difference of 3.22. This suggests that background information does matter.

#### Student Professional Approach Level

Through student professional approach-level indicator (Table 3), one can understand the scoring difference between the student and teacher. The teacher can discuss with students the difference and let students understand the teaching objectives the teacher values when conducting scoring in class. The figure in Table 3 is the percentage growth rate. The range of figure refers to Liu (2016); if it is 1+, it means that it is approaching 100% with the teacher; 0.95 means that the student reaches about 95% of the teacher's professional degree; 0.8 means that the student achieves about 80% of the teacher's professionalism. This figure is the same as the teacher's rating of 0 ~ 100. Observe the teacher and students' rating from a third-party perspective to obtain an objective professional value.


(1) $$\begin{array}{l} Students^{\prime }\;professional\;approach\;level\;\\ \;=\frac{Average\;score\;of\;peer\;assessment}{Score\;of\;teacher\;assessment} \end{array}$$

**Table 3:** Student professional approach table.

**Numerical range**	**Descriptions**
>1+	Students and teachers share the same comprehension approach
>0.95	Have a general understanding of the teaching objectives
>0.8	Have a slight difference in scores
>0.75	Can still mutually understand the way of scoring
>0.6	There are differences in mutual scoring approach
<0.6	Major intellectual gap between students and teachers

#### Student Self-Professional Growth Change

After students experienced assessment more than once, they can conduct comparison on the degree of difference based on the score data and understand their learning status according to the magnitude of the difference. Students can discuss whether the benchmark of scoring results can effectively improve the learning outcome and understand the accomplishment status of current curriculum and teaching objectives based on multiple records. Formula 2 can be used to calculate student professional growth change value, which can be applied to Table 4 to understand the student professional growth rate.


(2) $$\begin{array}{l} Students^{\prime }\;self-professional\;growth\;change\\ \;=1-\frac{nth\;assignment}{n-1th\;assignment} \end{array}$$

**Table 4:** Student self-professional growth change table.

**Numerical change**	**Changes in learning status**
>0.4	Significant progress from benchmark value
>0.2	Significant progress difference
0.0	Room for improvement
< -0.2	Moderate decline in progress
< -0.4	Significant decline from benchmark value
>0.4	Significant progress from benchmark value

### Analytical Assessment on Course Content

The five stages of the course content in this study are detailed in Table 5. Stage 1 is course planning. The English program code of the text interface is first changed into block programming or a set of block programming material is designed to allow easy learning for students. Stage 2 is to incorporate peer assessment into the course when it was appropriate. This is the learning stage for students using the development environment provided by Mit App Inventor 2. The reason for adopting Inventor 2 is that students can have easy access with a Google account. In addition, it is operated on the webpage, and no additional program download is required. Inventor 2 is a block-type programming, which is in line with the research direction of the study. Another purpose of this stage is to employ the self-developed material to observe whether students have improved their performance in programming through using block program. Stage 3 is the assessment stage. The tool employed is Zuvio. Zuvio was developed to strengthen students' and teachers' development capacity. The many functions it provides include peer assessment, teacher–student interaction, and student feedback. Zuvio also provides quantitative analysis; thus, this study mainly takes Zuvio to conduct peer assessment and student feedback. Stage 4 is data analysis. Software used is R; the reasons are described in sections Literature Review, Conceptual Framework, and Learning Effectiveness Analysis. In this stage, mainly the assessment data are used for analysis and integration to analyze students' learning status. The last stage is improvement which improves the course content based on the analytical data and consolidated data, including student feedback.

**Table 5:** Student self-professional growth change.

**Content of peer assessment teaching model**		
**Stage/theme**	**Tool**	**Content**
I. Course planning	None	1. Design the teaching material by changing text interface program into block program. 2. Incorporate peer assessment into grading system and adjust course content accordingly in a timely manner.
II. Student learning	MIT APP Inventor 2	1. Promote students' interest in learning programming by using block programming. 2. Encourage students to solve the problems independently through self-developed material and exercises.
II. Assessment	Zuvio	1. Guide students to understand the assessment criteria so that they learn how to score. 2. At the end of each assessment, students can give teachers their comments through Zuvio.
VI. Data analysis	Analytical software R	1. Find the teaching methods suitable for students by using R and other analytical software 2. Use statistical software to obtain the data and observe students' learning status.
V. Improvement	None	1. Use the results of analysis to improve teaching content. 2. Use the feedback function of Zuvio to understand students' learning status.

## Learning Effectiveness Analysis

The subjects of the study were the students in the mobile device development course. A total of 72 students divided into 18 groups participated in this research, and the students were grouped freely, with each team having four students. Experimental class (A) and control class (B) were conducted in this research. Experimental class included non-information major students (A1) and information major students (A2), and control class included non-information major students (B1) and information major students (B2). The analysis of students' learning effectiveness is given as follows.

### Relationship Between the Professional Level of the Teacher and Student Scoring

The scores of the midterm and final examinations were calculated by using formula 1, and the results are shown in Table 6. It can be seen that both the degree of approach between the midterm and final examinations was >0.9, indicating the difference between the students' and teacher's understanding of the scoring method is small. According to Table 7, the progress range of class A was 0.02, indicating the students have made slight improvement in grades, but there still existed room for improvement; the progress range of class B was 0.19, indicating students have made significant growth in the progress range.

**Table 6:** Teacher and student score approaching degree.

**Class**		**Score average**		**Approaching degree**	**Variations**
		**Peer**	**Teacher**		
A	Midterm	73.05	77.62	0.94	Have a general understanding of the teaching objective
	Final	74.82	79.34	0.94	Have a general understanding of the teaching objective
B	Midterm	69.93	74.73	0.93	Have a general understanding of the teaching objective
	Final	70.69	77.80	0.90	Have a general understanding of the teaching objective

**Table 7:** Student self-grow change.

**Class**	**Midterm Ave**.	**Final Ave**.	**Progress**	**Changes in learning status**
A	77.62	79.21	0.02	Students still have room for improvement.
B	61.00	73.08	0.19	Significant difference in making progress

**Figure 2:**
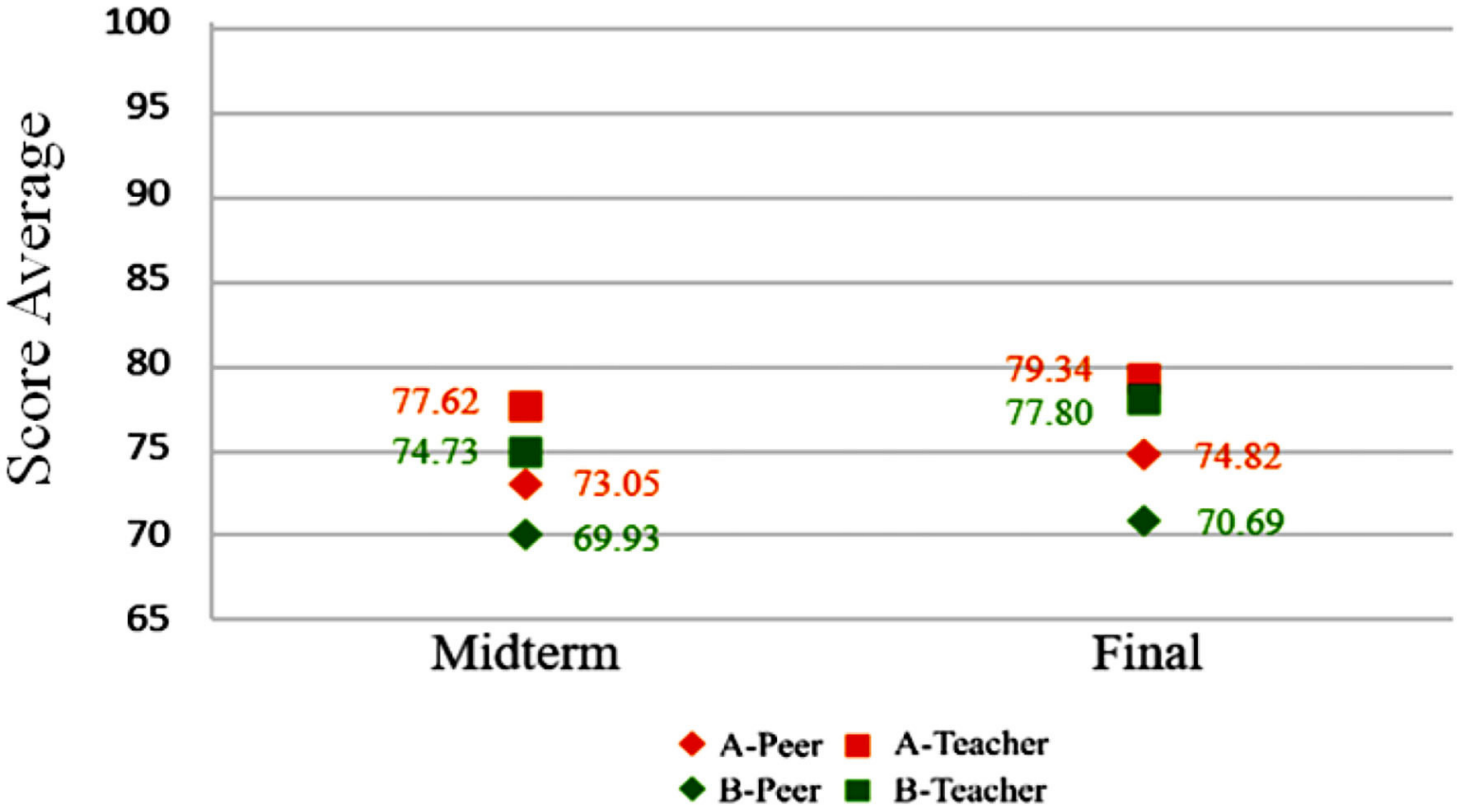
Teacher and student score approaching degree.

Figure 2 shows two sets of midterm and final examination scores for experimental group A and control group B. The scores are based on peer assessment and teacher's evaluation. Another score indicating cognitive differences between the two groups for midterm examination performance hovers around 0.93 and 0.94, suggesting the influence of unfamiliarity with the rubrics. By the final examination, cognitive difference of group B is narrowed down to 0.90. However, students in this group make no notable progress. By contrast, average scores in experimental group A are higher than those of midterm examination grades, while the cognitive difference stays at 0.94, showing learning progress and familiarity with the rubrics.

### Approaching Relationship Between Student and Teacher Scores

**Figure 3:**
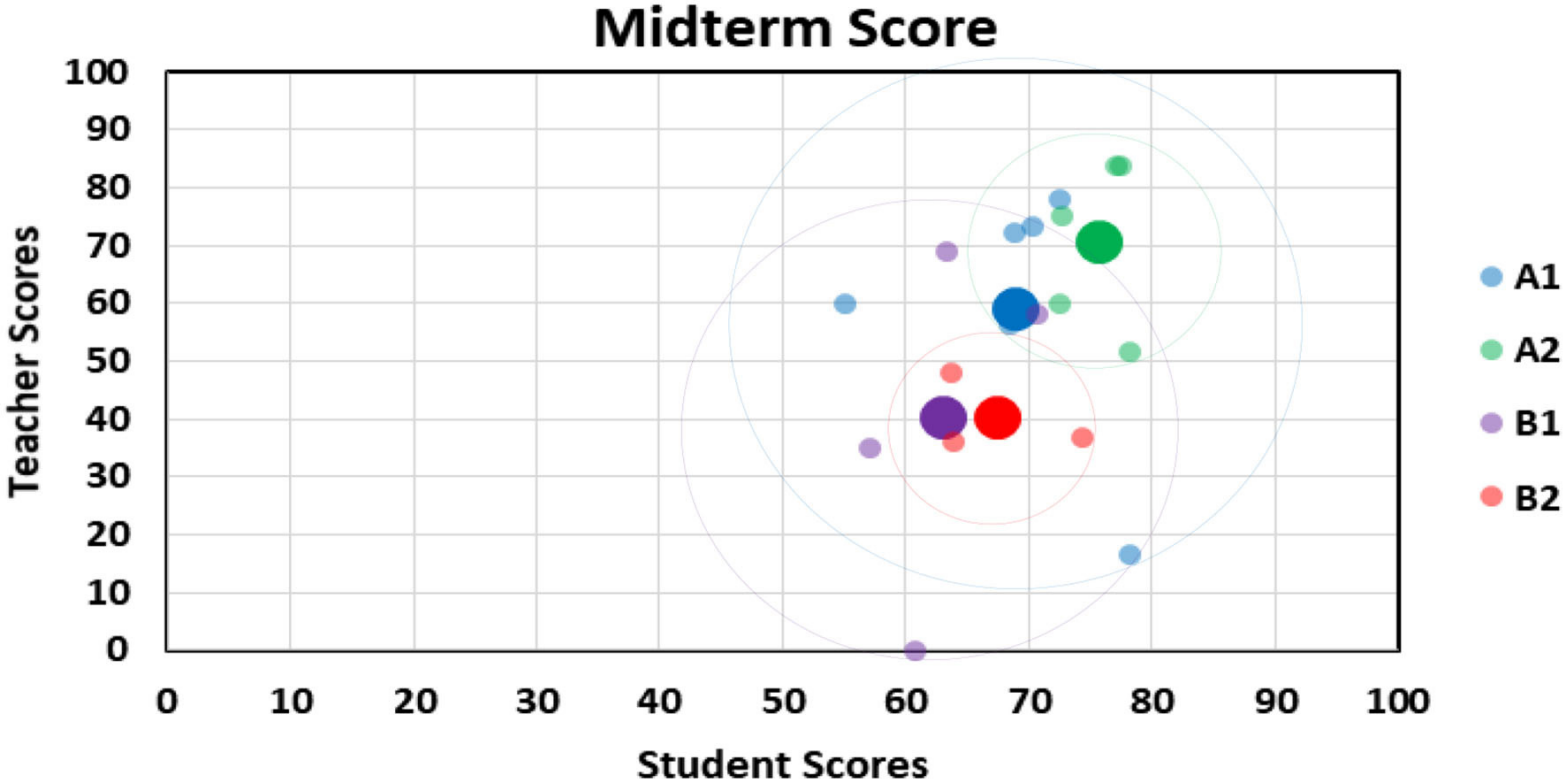
Midterm score approach: classes A1, A2, B1, and B2.

**Figure 4:**
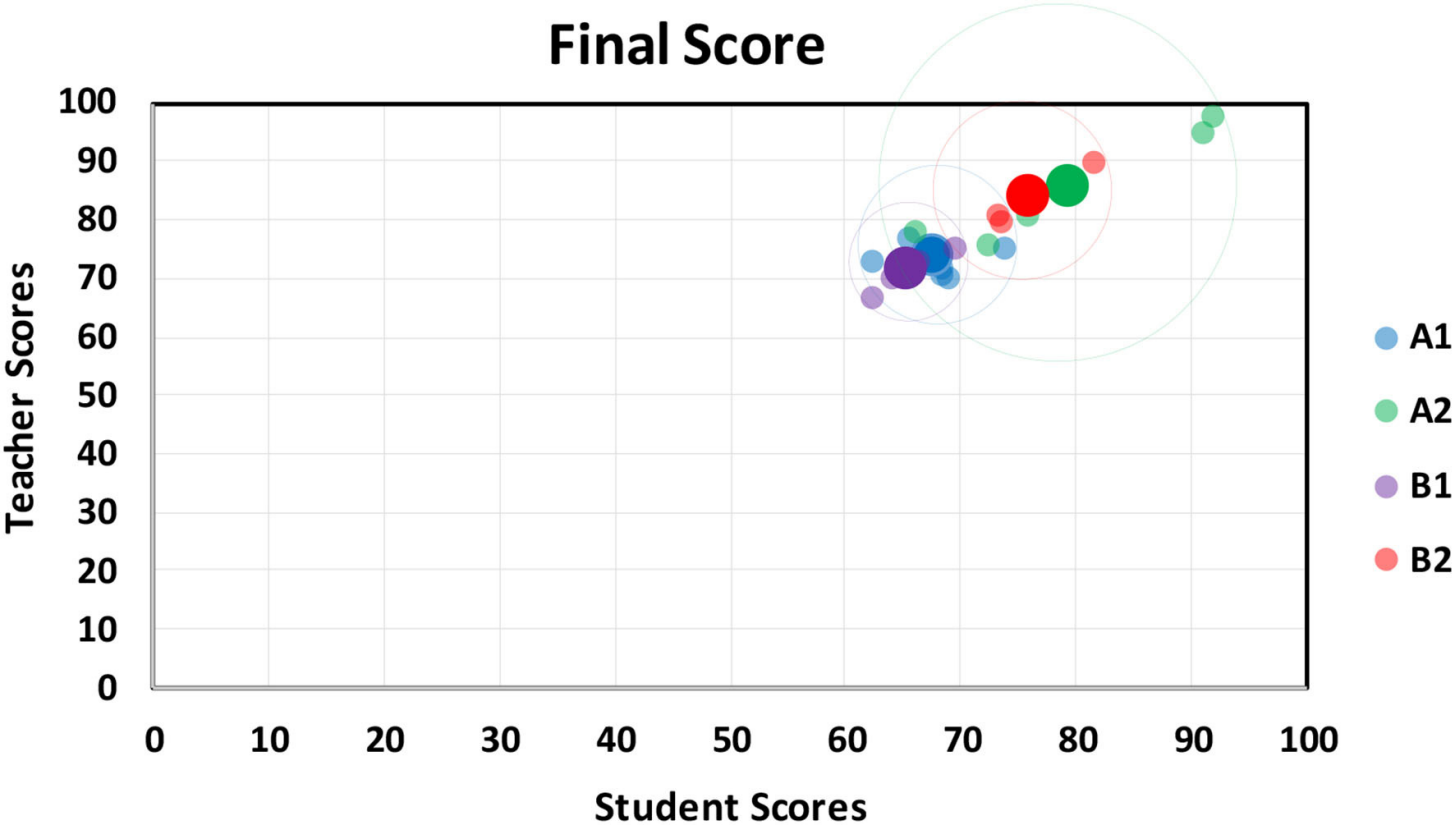
Final score approach: classes A1, A2, B1, and B2.

Teacher scores and student scores are presented in the distribution chart; the horizontal axis is the teacher score, and the vertical axis is the student score. Through the peer evaluation methods at the middle and end of the period, the range of the distribution points of the results is observed to understand the students' learning effectiveness. The progress rate is calculated by the aforementioned formula. Figures 3, 4, respectively, show the midterm and final examination grade distribution of the two classes, where the small point is the average score of the 18 groups, and the larger point is the overall average of the group. According to the original classification of the non-information major group (A1), the experimental class information major group (A2), the control class non-information major group (B1), and the experimental class information major group (B2), the average score of the information major group is higher than that of the non-information major group, and the scores of the experimental class are higher than those of the control class. It can be seen that when the experimental class uses the peer evaluation method, the students' learning status and results are better than the those using the traditional evaluation method of the control class.

Midterm examination results show the following findings: the average score of A2 and B2 students who have some knowledge of information science is higher than that of A1 and B1 peers who are first-time learners within the same class; the grade gap among learners in A2 and B2 is narrower, indicating that background knowledge of programming logic does make a difference.

Final examination results show a similar trend in average scores. Nevertheless, the grade gap among all students becomes narrower, with the average scores of the four groups increasing. It is clear proof that such a teaching method is well-received by students and that peer pressure has positive impacts. In other words, such innovative programming and peer assessment can enhance student performance.

**Figure 5:**
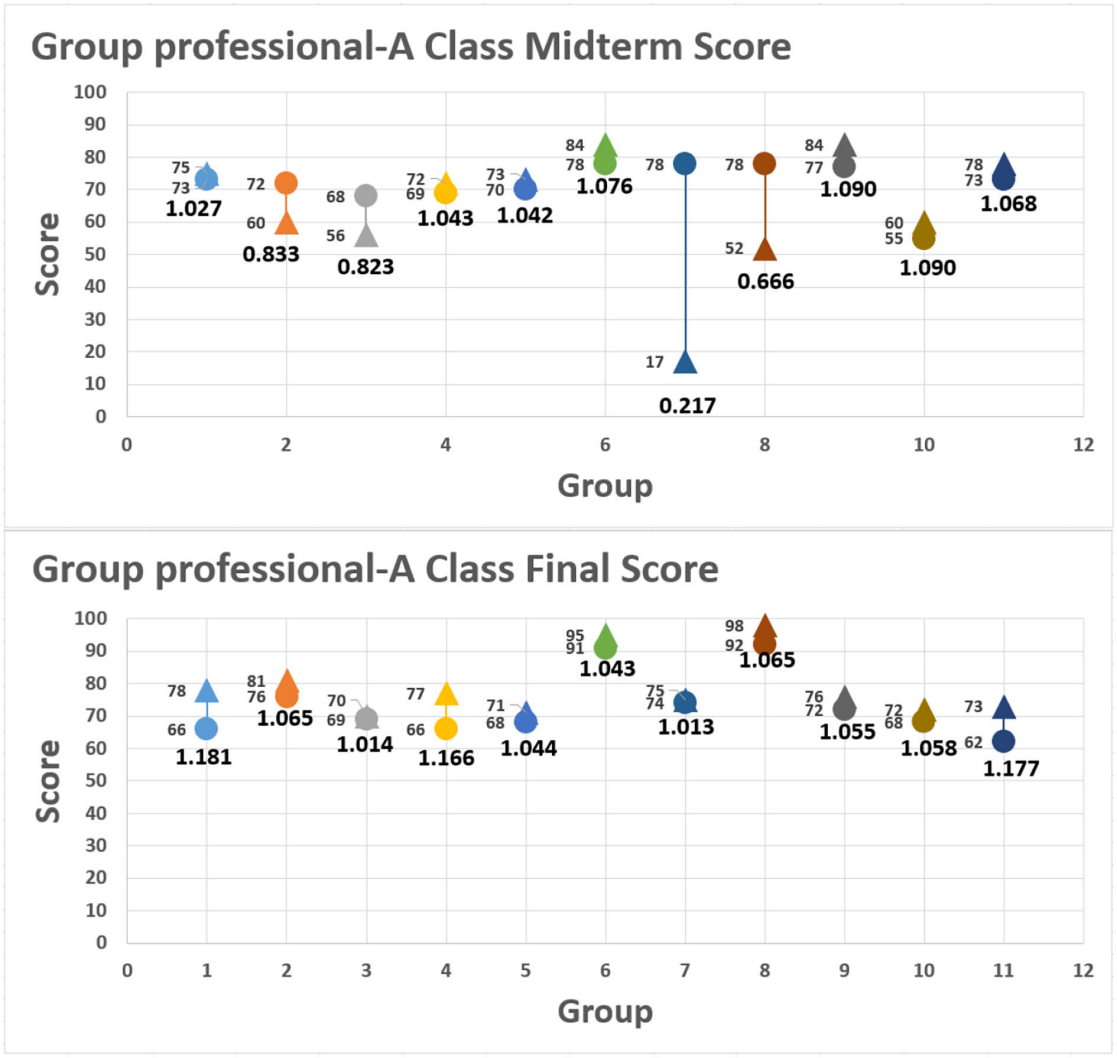
Group professional-level variation: class A.

**Figure 6:**
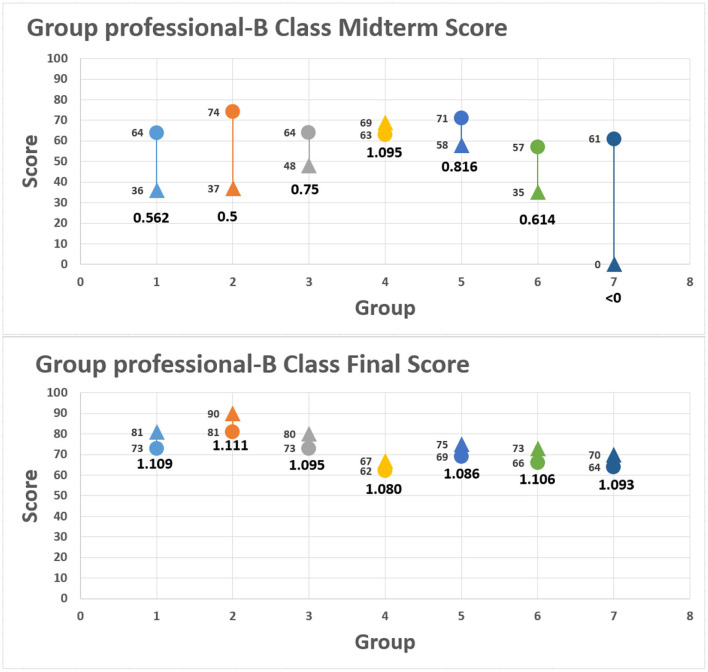
Group professional-level variation : class B.

Student scores and teacher scores were presented, respectively, in the distribution chart, with the horizontal axis as the student seat number and vertical axis as the scores given by the student and teacher. There were two types of graphs in the charts representing teacher or student scores, respectively. The round dot represents the score from peer assessment; the triangle represents the score from the teacher with the score indicated on the left. The figure below the graph represents the approaching degree between student and teacher scores, as is shown in Figures 5, 6. There were two classes in this study. The student numbers of the class are 46 for class A and 26 for class B. Each class was divided into several groups, with 11 groups in class A and 7 groups in class B. According to Figures and formula 1, it can be observed that the scores of both teacher and students in most groups were >1, indicating the students and teacher had the same understanding of the assessment criteria. A few groups did show a significant deviation from the teacher scores, for example, for group 7 of class A, the value calculated by the formula was only 0.217, which indicated that a significant gap existed in the judging score benchmark of the group and the teacher.

**Figure 7:**
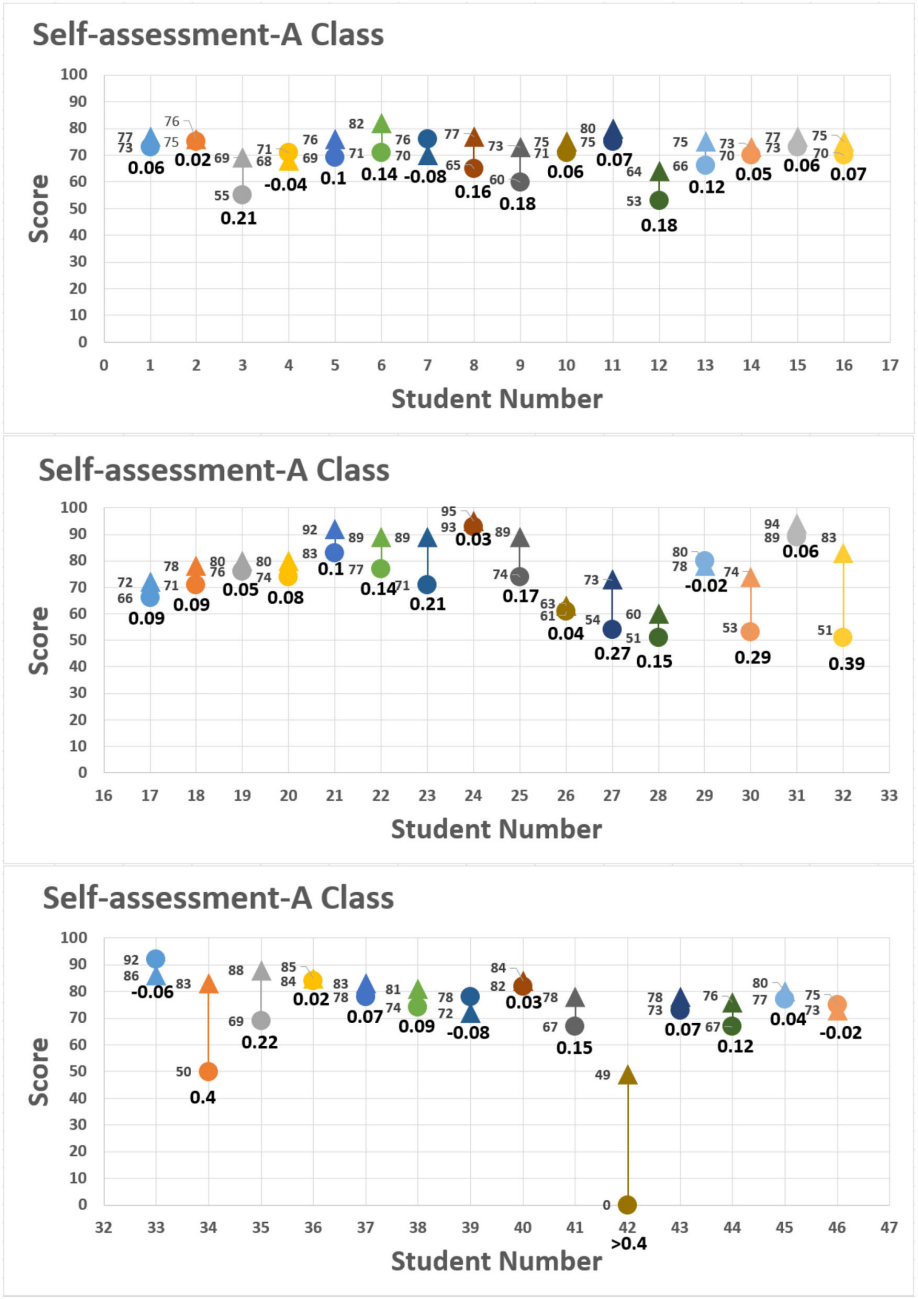
Self-assessment: class A.

**Figure 8:**
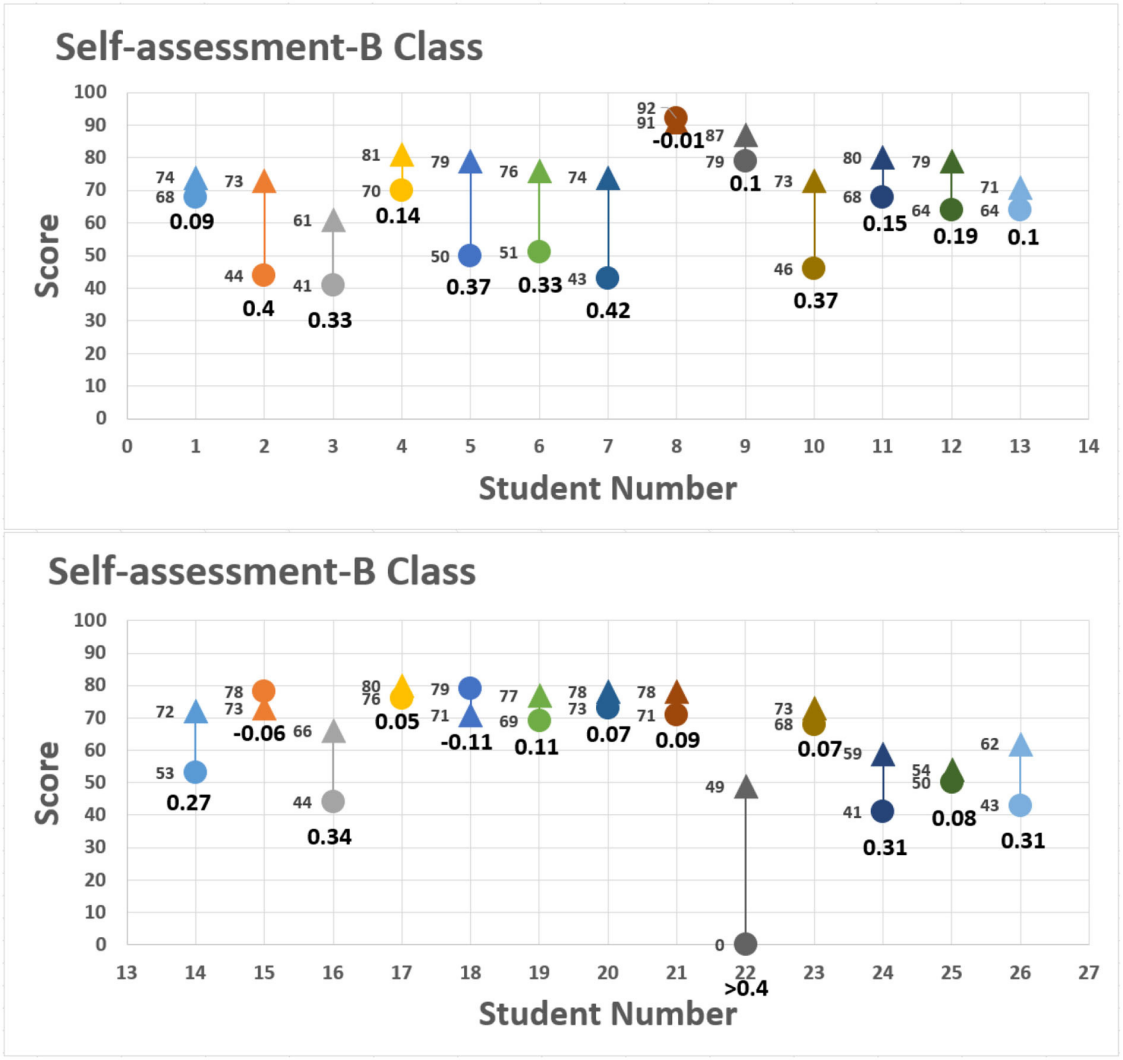
Self-assessment: class B.

The assessment also allowed students to substitute their assignment scores into formula 2 to measure their own progress. Students' self-evaluation rubrics are shown in Figures 7, 8, with the horizontal axis as the student seat number and vertical axis as the scores given by the students and teacher. The round dot represents the score of the first assignment; the triangle represents the score of the second assignment. The scores from both assignments are shown at the left of the distribution chart, and the value below indicate the range of progress or regression calculated by using formula 2. The final self-assessment results showed most students demonstrated slight degree of positive growth. For example, No. 7 of class B made the highest growth rate of 0.42 in the self-assessment, progressing from 43 points to 74 points, as 0.42 > 0.4 representing substantial growth in accomplishing the assignment. The score of No. 22 (Figures 7, 8) among group 7 (Figures 3, 6) in the control class is given for peer evaluation at the end of the period, but the report was not uploaded to the Zuvio platform. According to the regulations, the teacher cannot score it, so it is 0 point.

The study is fully aware that students who have no background knowledge of programming find it challenging to familiarize themselves with the English-language interface and syntax. To address this challenge, this research introduces an innovative teaching method that is reinforced by peer assessment rubrics and interactive instruction platforms and explores the potential of peer pressure and block programming. Through discussion and sharing, students become more adept at articulating ideas, solving problems, and cooperating with team members.

### Discussion

The study introduces an innovative teaching method that is reinforced by peer assessment rubrics and interactive instruction platforms, and explores the potential of peer pressure and block programming. Grades of experimental and control groups are analyzed, indicating that such a method does improve programming learning performance in the traditional educational context. Thus, the study suggests that college teachers and programming trainers adopt such a method to turn peer pressure into enhanced performance.

It is not surprising to find that students who have no prior exposure to programming do not fare well in the traditional educational context, which in turn undermines their motivation and confidence. Therefore, introducing more learning strategies and platforms should not aim to engage students' interest but to motivate them to overcome difficulties and improve learning outcomes.

**Figure 9:**
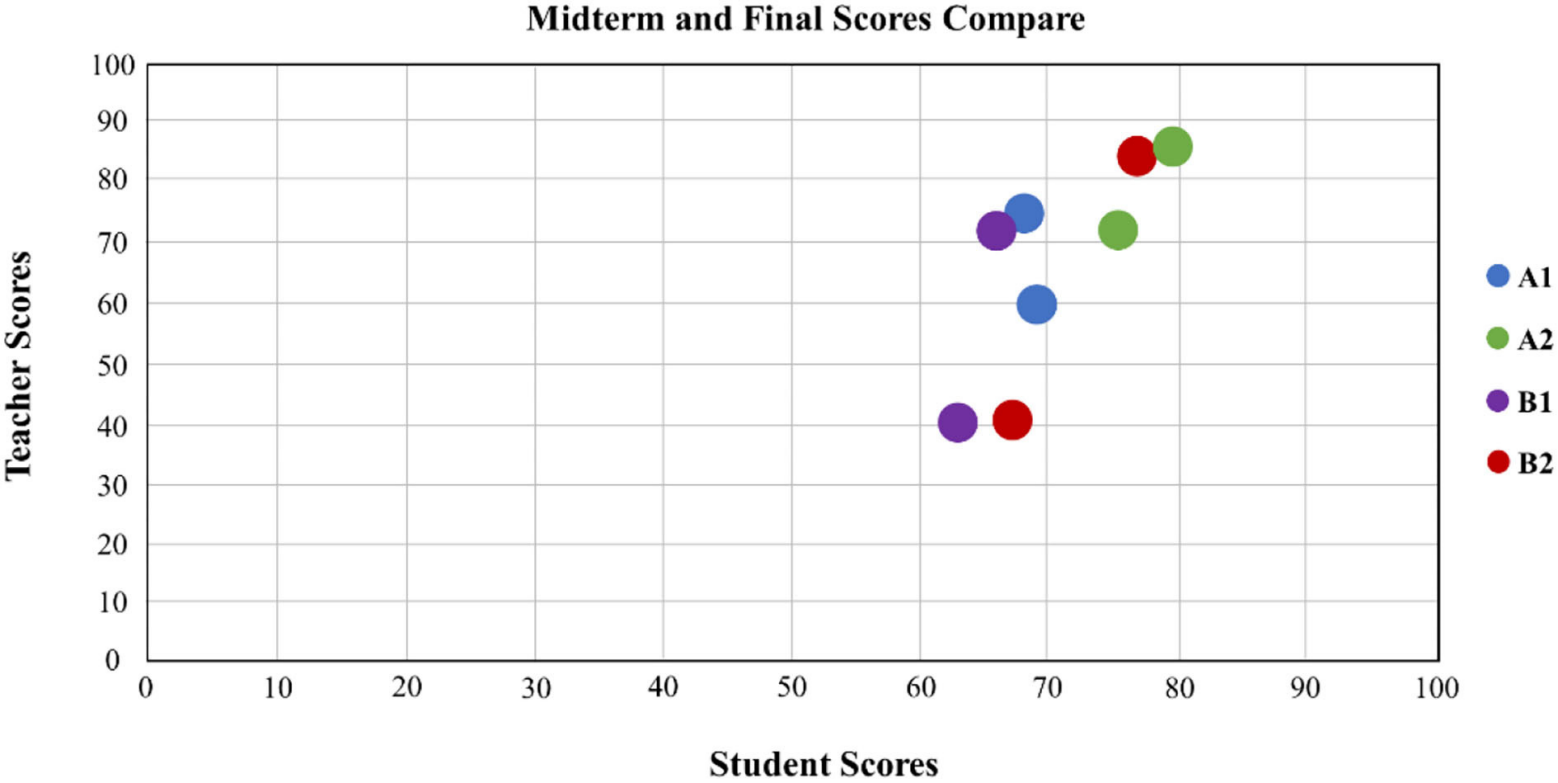
Midterm and final examination score comparison: classes A1, A2, B1, and B2.

Figure 9 shows cognitive differences among A1, A2, B1, and B2 for midterm and final examination scores. Average scores based on peer assessment and teacher's evaluation rise to some extent. Cognitive differences for B1 and B2 are higher than those of A1 and A2, suggesting the former two groups' unfamiliarity with the rubrics. Between A1 and A2, with the former knowing nothing about programming and the latter having some background knowledge, cognitive differences are smaller, which can be attributed to the familiarity with peer assessment rubrics.

The experimentation results indicate that grouping is not a differentiating factor because both groups showed signs of improvement. Nevertheless, it is worth noting that when engaging in peer assessment at the initial stage, students with no background information were more productive than those taught by the traditional method, suggesting that peer pressure is a motivator and enhancer of learning outcomes. In the second stage, the learners also delivered better performance than the control group, suggesting that peer pressure, companionship, and instruction do improve beginners' performance.

The instruction of computational thinking begins with its definition and teaching method. The first step is to differentiate between mathematical thinking and computational thinking. The latter covers four aspects: data application, modeling and simulation, problem-solving, and systemic thinking. This research focuses on the latter two. To this end, the authors examine related studies, as well as 101 block-based programming environments, of which 46 are analyzed to compare different designs that support the transition to text-based programming. Totally, 10 principles of tool construction are provided. An innovative teaching method is created to facilitate the learning of block-based programming and to improve learning performance.

## Conclusion

The study analyzes the data obtained from the procedures, and peer assessment is a distinguishing feature of it. Class A and B students reported that high-school or vocational school courses related to information science did influence their performance. However, this perceived difference is not validated by either peer assessment or instructor evaluation. Both scores are higher than 0.90, suggesting that students and the instructor have a similar understanding of the course's objectives. One possible explanation is that the familiarization with the peer assessment rubrics gives students a deeper understanding of what they are expected to achieve. As for the indicator of self-improvement, the difference among class A students between midterm and final examination results is 0.02, suggesting considerable room for growth. The difference for class B students is 0.19, indicating significant progress. Therefore, the familiarization with the peer assessment rubrics has an influence on the scoring distribution of instructor evaluation and peer review. The dispersion for the midterm examination is more scattered. In the final examination, the dispersion is more clustered, indicating that benchmark values between peer assessment and instructor evaluation are close. It also shows that students and their peers achieved a general consensus on how to gain the specialized knowledge and how to gauge peer assessment.

The study incorporated peer evaluation mechanism in a classroom setting to understand whether the new instruction model was beneficial to student learning in higher education. Exploring role identity within the social identity theory enables us to understand better how the role or identity impacts engagement, retention, and persistence. The study employed Zuvio for peer evaluation. The results showed that in classes A and B, for those who majored in information science in regular high school or vocational high school, the variation in professional approach degrees was limited. Thus, in terms of programming, the variation between student scores and teacher scores was limited. The students' professional approach degree value was higher than 0.90, which indicated that students and teacher had similar understanding of the teaching objectives. On the other hand, when analyzing the self-professional growth changes of classes A and B, it was found that the self-professional growth change range for class A was 0.02, which indicated that there was still room for improvement in learning growth. Class B demonstrated a 0.19 change range. In other words, all students have sustainable development in their reflective learning. In learning, there was a significant progress in the final than in the midterm examination. In the approaching relationship between the teacher and students, both teacher scores and student scores were presented in the distribution chart. Through the dot distribution, it can be found that the spread was more scattered, indicating a larger variation between teacher and student scores. In final assessment, the spread was more intense, indicating students' peer assessments already have a similar perspective with the teacher assessment, and they shared the same understanding on professional knowledge and assessment methods.

The results show that peer evaluation reaches a sustainable development goal in programming education. Non-information major students within peer evaluation have higher effective learning in programming than those in traditional teaching methods in the beginning stage, which means that students are more likely to learn programming because of peer pressure. Non-information major students also show higher learning effectiveness in the second stage than those in the control class, which means that under the pressure and guidance of peers, peer assessment teaching makes students more attractive to programming courses.

The study designed an innovative teaching method whose effectiveness is examined by analyzing the grades of experimental and control groups, with a greater emphasis on the learning performance of the former. Students in this group have no prior exposure to programming. Nevertheless, final examination results indicate that their performance is improved, better than that of their counterparts in the control group. In other words, the innovative teaching method does make a difference.

Given that programming logic and similar courses are attached greater importance by higher education institutions, how to replace the traditional teaching method with innovative ones and ensure the latter suits the current learning environment deserves serious attention. The methodology and innovative teaching method designed by this research can provide some inspiration for college teachers and programming trainers who can guide students to cope with peer pressure, and improve learning performance.

## Limitation and Future Work

The study has some limitations: (a) the course spans only one semester; (b) few classes and learners engage in the experiment; and (c) the division of category or group needs to be more precise. Thus, statistical accuracy needs to be increased. If this experiment is run for years to have more data collected, statistical accuracy will be ensured, and clear patterns be found.

Based on the conclusion, the authors suggest that peer assessment, an innovative teaching method, should be adopted for module-based course instruction. Compared to the traditional teaching style, peer pressure and comparison motivate students to do better and engage their learning interest in programming. But the one-size-fits-all approach should be avoided. The peer assessment rubrics should take into account learners' characteristics. The fundamental criterion should be whether such assessment maximizes learning outcomes.

## Data Availability Statement

The original contributions presented in the study are included in the article/supplementary material, further inquiries can be directed to the corresponding authors.

## Ethics Statement

Ethical review and approval was not required for the study on human participants in accordance with the local legislation and institutional requirements. Written informed consent from the [patients/participants OR patients/participants legal guardian/next of kin] was not required to participate in this study in accordance with the national legislation and the institutional requirements.

## Author Contributions

T-CH and Y-HC: conceptualization and data curation. T-LC: methodology. T-LC and C-YC: software. T-CH, Y-HC, and C-CC: validation. T-CH: formal analysis, investigation, and funding acquisition. T-CH and C-CC: resources, supervision, and project administration. T-LC and T-CH: writing—original draft preparation, writing—review and editing, and visualization. All authors have read and agreed to the published version of the manuscript.

## Funding

This research was supported by the Fundamental Research Funds for the Central Universities (Grant Number 3213049408), projects under MOST 108-2221-E-390-005 and MOST 108-2622-E-390-002-CC3. This research was also supported by the major education and teaching reform projects in Fujian undergraduate colleges and universities in 2019 (grant FBJG20190284).

## Conflict of Interest

The authors declare that the research was conducted in the absence of any commercial or financial relationships that could be construed as a potential conflict of interest.

## Publisher's Note

All claims expressed in this article are solely those of the authors and do not necessarily represent those of their affiliated organizations, or those of the publisher, the editors and the reviewers. Any product that may be evaluated in this article, or claim that may be made by its manufacturer, is not guaranteed or endorsed by the publisher.
